# Correction: A machine learning approach identifies distinct early-symptom cluster phenotypes which correlate with hospitalization, failure to return to activities, and prolonged COVID-19 symptoms

**DOI:** 10.1371/journal.pone.0349084

**Published:** 2026-05-07

**Authors:** The PLOS One Staff

## Notice of Republication

This article was republished on April 27, 2026, to correct an error in the author list. Stephanie A. Richard is not included in the author byline. Stephanie A. Richard should be listed as the 31st author, and her affiliations are #1 and #2: Infectious Disease Clinical Research Program, Department of Preventive Medicine and Biostatistics, Uniformed Services University of the Health Sciences, Bethesda, Maryland, United States of America, and Henry M. Jackson Foundation for the Advancement of Military Medicine, Inc., Bethesda, Maryland, United States of America, respectively. The contributions of this author are as follows: Conceptualization/Formal analysis/Investigation/Methodology/Project administration/Resources/Supervision/Writing – original draft/Writing – review & editing. The publisher apologizes for the error. Please download this article again to view the correct version. The originally published, uncorrected article and the republished, corrected articles are provided here for reference.

## Supporting information

S1 File Originally published, uncorrected article. (PDF)

S2 File Republished, corrected article. (PDF)
