# Supplementary material for: A machine learning approach identifies distinct early-symptom cluster phenotypes which correlate with hospitalization, failure to return to activities, and prolonged COVID-19 symptoms
Source: PLoS One. 2023 Feb 9;18(2):e0281272. doi: 10.1371/journal.pone.0281272 (PMC9910657; doi:10.1371/journal.pone.0281272)
Supplement: S1 File — (DOCX) [file pone.0281272.s001.docx]

**Supporting Information**

**A machine learning approach identifies distinct early-symptom cluster phenotypes which correlate with hospitalization, failure to return to activities, and prolonged COVID-19 symptoms**

*Epsi et al.,*

**Supplementary Figure**

S1 Figure: Flowchart diagram of included participants

**Supplementary Tables**

S1 Table. Clinical and demographic characteristics of military health system beneficiaries by responder and non-responder

S2 Table: Crude and adjusted association of covariates with hospitalization in 1273 SARS-CoV-2 infections in Military Health System beneficiaries.

S3 Table: Crude and adjusted association of covariates with failure to return to usual activities in 1273 SARS-CoV-2 infections in Military Health System beneficiaries

S4 Table: Crude and adjusted association of covariates with failure to return to usual health in 1273 SARS-CoV-2 infections in Military Health System beneficiaries

S5 Table. Clinical and demographic characteristics of 529 military health system beneficiaries who filled out surveys at six-months post-symptom onset by identified clusters

S6 Table. Prolonged COVID-19 symptoms of 529 Military Health System beneficiaries by identified clusters at six-months

S7 Table: Univariable and multivariable models to identify correlates of long COVID among those reported six months persistent symptoms

S8 Table: Univariable and multivariable models to compare adjusted log10 based CRP levels in identified clusters

S9 Table: Univariable and multivariable models to compare adjusted log10 based IL-6 levels in identified clusters

**Supplementary Figure:**

| 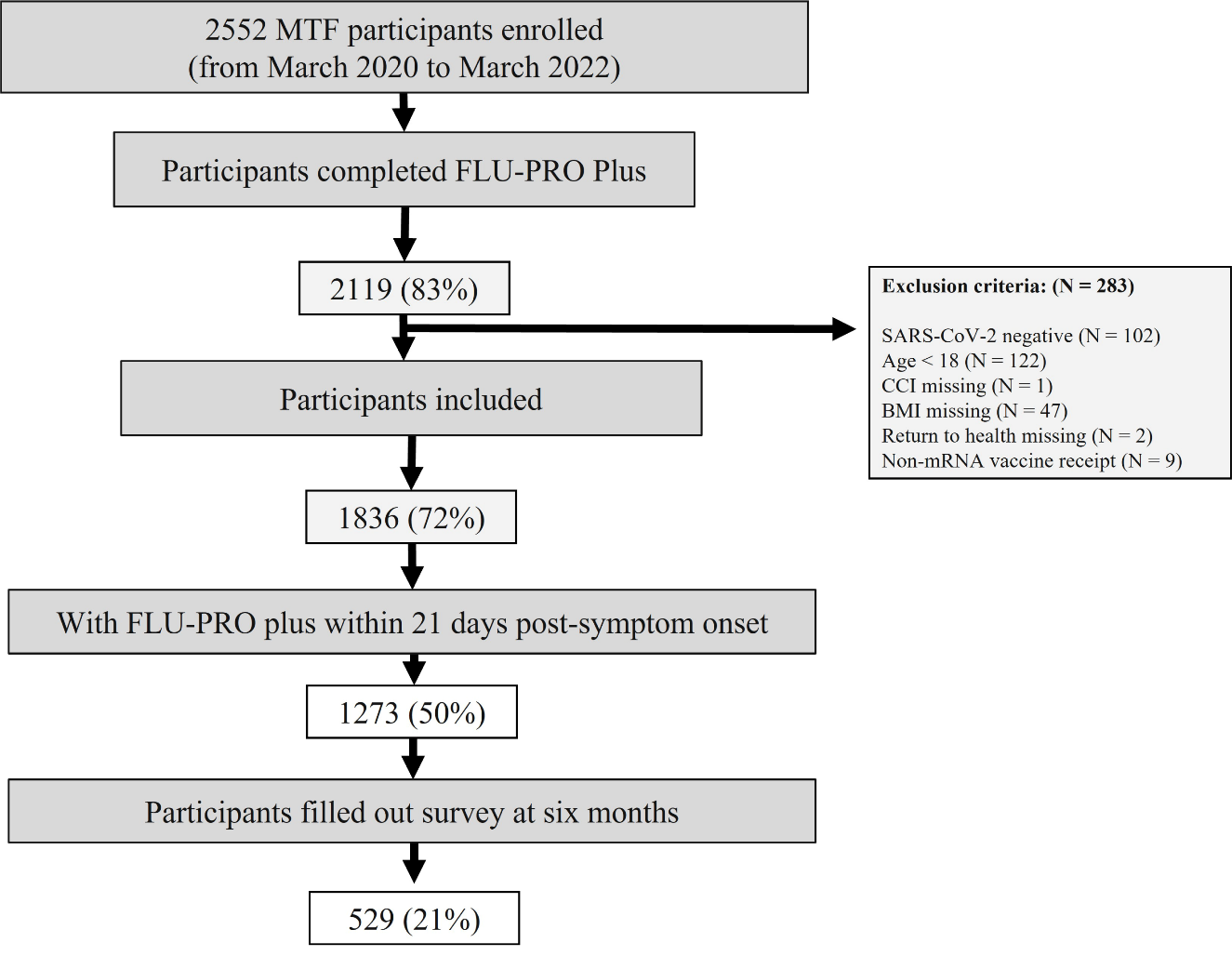 |
| --- |
| **S1 Figure:** Flowchart diagram of included participants |

**Supplementary Tables:**

**S1 Table. Clinical and demographic characteristics of military health system beneficiaries by responder and non-responder**

|  | **Responder*^a^* (N=1273)** | **Non-responder*^b^* (N=433)** | **Total (N=1706)** | ***P* value*^c^*** |
| --- | --- | --- | --- | --- |
| **Age group** |  |  |  | < 0.01 |
| <18 | 0 (0.0%) | 86 (19.9%) | 86 (5.0%) |  |
| 18-44 | 766 (60.2%) | 182 (42.0%) | 948 (55.6%) |  |
| 45-64 | 384 (30.2%) | 106 (24.5 %) | 490 (28.7%) |  |
| 65+ | 123 (9.7%) | 59 (13.6%) | 182 (10.7%) |  |
| **Sex** |  |  |  | 0.52 |
| Female | 531 (41.7%) | 173 (40.0%) | 704 (41.3%) |  |
| Male | 742 (58.3%) | 260 (60.0%) | 1002 (58.7%) |  |
| **Race/Ethnicity** | |  |  | < 0.01 |
| Black | 158 (12.4%) | 76 (17.6%) | 234 (13.7%) |  |
| Hispanic or Latino | 333 (26.2%) | 113 (26.1%) | 446 (26.1%) |  |
| Other | 125 (9.8%) | 56 (12.9%) | 181 (10.6%) |  |
| White | 657 (51.6%) | 188 (43.4%) | 845 (49.5%) |  |
| **Charlson Comorbidity Index** | | |  | 0.03 |
| 0 | 793 (62.3%) | 262 (60.5%) | 1055 (61.8%) |  |
| 1-2 | 302 (23.7%) | 92 (21.2%) | 394 (23.1%) |  |
| 3-4 | 107 (8.4%) | 37 (8.5%) | 144 (8.4%) |  |
| >5 | 71 (5.6%) | 42 (9.7%) | 113 (6.6%) |  |
| **Severity** |  |  |  | < 0.01 |
| Hospitalized | 243 (19.1%) | 127 (29.3%) | 370 (21.7%) |  |
| Outpatient | 1030 (80.9%) | 306 (70.7%) | 1336 (78.3%) |  |
| **Variants** |  |  |  | < 0.01 |
| Alpha | 38 (3.0%) | 15 (3.5%) | 53 (3.1%) |  |
| Delta | 159 (12.5%) | 44 (10.2%) | 203 (11.9%) |  |
| Omicron | 86 (6.8%) | 12 (2.8%) | 98 (5.7%) |  |
| Other***^d^*** | 551 (43.3%) | 99 (22.9%) | 650 (38.1%) |  |
| Unknown*^e^* | 439 (34.5%) | 205 (47.3%) | 644 (37.7%) |  |
| **Body Mass Index** | |  |  | 0.3 |
| Normal | 257 (20.2%) | 55 (17.8%) | 312 (19.7%) |  |
| Overweight | 488 (38.3%) | 109 (35.3%) | 597 (37.7%) |  |
| Obese | 313 (24.6%) | 81 (26.2%) | 394 (24.9%) |  |
| Severely Obese | 215 (16.9%) | 64 (20.7%) | 279 (17.6%) |  |
| **Military Status** | |  |  | < 0.01 |
| Active duty | 634 (49.8%) | 143 (33.0%) | 777 (45.5%) |  |
| Civilian | 0 (0.0%) | 1 (0.2%) | 1 (0.1%) |  |
| Dependent | 344 (27.0%) | 182 (42.0%) | 526 (30.8%) |  |
| Retired military | 295 (23.2%) | 107 (24.7%) | 402 (23.6%) |  |
| **Department of Defense affiliation** | | |  | 0.69 |
| Air Force | 217 (17.0%) | 73 (16.9%) | 290 (17.0%) |  |
| Army | 585 (46.0%) | 195 (45.0%) | 780 (45.7%) |  |
| Coast Guard | 11 (0.9%) | 2 (0.5%) | 13 (0.8%) |  |
| Marines | 77 (6.0%) | 33 (7.6%) | 110 (6.4%) |  |
| Navy | 358 (28.1%) | 118 (27.3%) | 476 (27.9%) |  |
| Other*^f^* | 25 (2.0%) | 12 (2.8%) | 37 (2.2%) |  |
| **SARS-CoV-2 test** | |  |  | < 0.01 |
| Negative | 0 (0.0%) | 58 (13.7%) | 58 (3.4%) |  |
| Positive | 1273 (100.0%) | 364 (86.3%) | 1637 (96.6%) |  |
| **Vaccine** |  |  |  | < 0.01 |
| Johnson & Johnson | 0 (0.0%) | 9 (2.1%) | 9 (0.5%) |  |
| Moderna | 55 (4.3%) | 45 (10.4%) | 100 (5.9%) |  |
| Pfizer | 304 (23.9%) | 216 (49.9%) | 520 (30.5%) |  |
| Unvaccinated | 914 (71.8%) | 163 (37.6%) | 1077 (63.1%) |  |
| **Vaccine breakthrough cases** | | |  | < 0.01 |
| No | 945 (74.2%) | 376 (86.8%) | 945 (71.1%) |  |
| Yes | 328 (25.8%) | 57 (13.2%) | 385 (28.9%) |  |
| **Moderate to severe symptoms at six-months*^g^*** | | | | 0.17 |
| No | 480 (90.7%) | 52 (96.3%) | 532 (91.3%) |  |
| Yes | 49 (9.3%) | 2 (3.7%) | 51 (8.7%) |  |
| *^a^Responder those who filled out FLU-PRO Plus survey and included in the analysis (Table 1, S1 Figure)* | | | | |
| *^b^Non-responder those who did not filled out FLU-PRO Plus survey* | | | | |
| *^c^n x k Fisher’s exact test* | | | | |
| *^d^Other variants: Beta, Eta, Epsilon, Gamma, Iota, Theta* | | | | |
| *^e^Sequence failed to be mapped to a lineage* | | | | |
| *^f^Other DOD affiliation includes National Guard, National Oceanic and Atmospheric Administration, US Public Health Service, and missing affiliations.* | | | | |
| *^g^Subset of participants who filled out surveys at six-months post-symptom onset^.^* | | | | |

| **S2 Table:** **Crude and adjusted association of covariates with hospitalization in 1273 SARS-CoV-2 infections in** **Military Health System beneficiaries.** | | | | |
| --- | --- | --- | --- | --- |
| **Covariates** | **Unadjusted risk ratio (95% CI)** | ***P* value** | **Adjusted risk ratio^a^ (95% CI)** | ***P* value** |
| Sensory Cluster^b^ | 0.88 (0.6 to 1.31) | 0.54 | 1.02 (0.68 to 1.53) | 0.92 |
| Respiratory/Systemic Cluster^b^ | 3.05 (2.22 to 4.2) | <0.01 | 2.24 (1.61 to 3.12) | <0.01 |
| Age group: 45-64 | 4.43 (3.26 to 6.02) | <0.01 | 2.12 (1.37 to 3.27) | <0.01 |
| Age group: 65+ | 5.59 (3.86 to 8.11) | <0.01 | 2.21 (1.26 to 3.87) | 0.01 |
| Sex: Male | 1.28 (0.99 to 1.67) | 0.06 | 1.14 (0.87 to 1.48) | 0.35 |
| Race: Black | 1.25 (0.75 to 2.09) | 0.39 | 0.89 (0.53 to 1.51) | 0.67 |
| Race: Hispanic | 1.22 (0.77 to 1.93) | 0.39 | 0.94 (0.59 to 1.5) | 0.79 |
| Race: White | 0.82 (0.52 to 1.27) | 0.37 | 0.66 (0.42 to 1.04) | 0.07 |
| CCI: 1-2 | 3.67 (2.69 to 5.01) | <0.01 | 1.55 (1.01 to 2.38) | 0.04 |
| CCI: 3-4 | 4.69 (3.2 to 6.87) | <0.01 | 2.11 (1.27 to 3.5) | <0.01 |
| CCI: ≥5 | 6.08 (4.07 to 9.07) | <0.01 | 2.6 (1.48 to 4.57) | <0.01 |
| Overweight | 2.18 (1.24 to 3.83) | 0.01 | 1.43 (0.81 to 2.54) | 0.22 |
| Obese | 4.43 (2.56 to 7.69) | <0.01 | 2.43 (1.38 to 4.26) | <0.01 |
| Severely obese | 6.77 (3.91 to 11.73) | <0.01 | 3.14 (1.78 to 5.54) | <0.01 |
| mRNA vaccine receipt | 0.31 (0.2 to 0.46) | <0.01 | 0.42 (0.28 to 0.64) | <0.01 |
| Days post-symptom onset | 1.05 (1.02 to 1.07) | <0.01 | 1.02 (0.99 to 1.04) | 0.19 |
| AIC |  |  | 1052.10 |  |
| BIC |  |  | 1139.64 |  |
| Num. obs. | 1273 |  | 1273 |  |
| *^a^Adjusted for age group, sex, race, comorbidity, obesity, vaccine receipt, and days post symptom onset* | | | | |
| *^b^Ref: Nasal Cluster* | | | | |
| *CCI = Charlson Comorbidity Index* | |  |  |  |
| *mRNA vaccines = Pfizer/BioNTech-BNT162b2, Moderna mRNA‐1273* | | | | |

| **S3 Table:** **Crude and adjusted association of covariates with failure to return to usual activities in 1273 SARS-CoV-2 infections in Military Health System beneficiaries** | | | | |
| --- | --- | --- | --- | --- |
| **Covariates** | **Unadjusted risk ratio (95% CI)** | ***P* value** | **Adjusted risk ratio^a^ (95% CI)** | ***P* value** |
| Sensory Cluster^b^ | 1.3 (1 to 1.68) | 0.05 | 1.22 (0.93 to 1.59) | 0.15 |
| Respiratory/Systemic Cluster^b^ | 1.83 (1.43 to 2.35) | <0.01 | 1.53 (1.18 to 1.97) | <0.01 |
| Age group: 45-64 | 1.59 (1.29 to 1.97) | <0.01 | 1.3 (0.95 to 1.78) | 0.1 |
| Age group: 65+ | 1.77 (1.32 to 2.39) | <0.01 | 1.44 (0.91 to 2.27) | 0.12 |
| Sex: Male | 1.06 (0.87 to 1.29) | 0.57 | 1.07 (0.87 to 1.31) | 0.54 |
| Race: Black | 1.08 (0.72 to 1.62) | 0.71 | 1 (0.66 to 1.5) | 0.99 |
| Race: Hispanic | 1.25 (0.88 to 1.78) | 0.2 | 1.14 (0.8 to 1.63) | 0.46 |
| Race: White | 0.78 (0.55 to 1.1) | 0.15 | 0.76 (0.54 to 1.07) | 0.12 |
| CCI: 1-2 | 1.51 (1.2 to 1.89) | <0.01 | 1.12 (0.81 to 1.54) | 0.5 |
| CCI: 3-4 | 1.53 (1.09 to 2.13) | 0.01 | 1.18 (0.76 to 1.82) | 0.46 |
| CCI: ≥5 | 2.14 (1.52 to 3.01) | <0.01 | 1.59 (0.99 to 2.57) | 0.06 |
| Overweight | 0.92 (0.68 to 1.23) | 0.57 | 0.78 (0.58 to 1.05) | 0.11 |
| Obese | 1.34 (0.99 to 1.8) | 0.06 | 1.06 (0.78 to 1.44) | 0.7 |
| Severely obese | 1.64 (1.2 to 2.23) | <0.01 | 1.17 (0.85 to 1.62) | 0.33 |
| mRNA vaccine receipt | 0.45 (0.34 to 0.59) | <0.01 | 0.51 (0.39 to 0.68) | <0.01 |
| Days post-symptom onset | 0.99 (0.97 to 1.01) | 0.39 | 0.98 (0.96 to 1) | 0.04 |
| AIC |  |  | 1657.91 |  |
| BIC |  |  | 1745.44 |  |
| Num. obs. | 1273 |  | 1273 |  |
| *^a^Adjusted for age group, sex, race, comorbidity, obesity, vaccine receipt, and days post symptom onset* | | | | |
| *^b^Ref: Nasal Cluster* |  |  |  |  |
| *CCI = Charlson Comorbidity Index* | |  |  |  |
| *mRNA vaccines = Pfizer/BioNTech-BNT162b2, Moderna mRNA‐1273* | | | | |
|  | |  |  |  |

| **S4 Table: Crude and adjusted association of covariates with failure to return to usual health in 1273 SARS-CoV-2 infections in Military Health System beneficiaries** | | | | |
| --- | --- | --- | --- | --- |
| **Covariates** | **Unadjusted risk ratio (95% CI)** | ***P* value** | **Adjusted risk ratio^a^ (95% CI)** | ***P* value** |
| Sensory Cluster^b^ | 1.19 (0.97 to 1.46) | 0.1 | 1.13 (0.91 to 1.39) | 0.28 |
| Respiratory/Systemic Cluster^b^ | 1.38 (1.12 to 1.69) | <0.01 | 1.22 (0.99 to 1.5) | 0.07 |
| Age group: 45-64 | 1.36 (1.14 to 1.63) | <0.01 | 1.34 (1.03 to 1.73) | 0.03 |
| Age group: 65+ | 1.32 (1 to 1.72) | 0.05 | 1.36 (0.91 to 2.04) | 0.13 |
| Sex: Male | 0.9 (0.76 to 1.06) | 0.19 | 0.9 (0.76 to 1.06) | 0.22 |
| Race: Black | 1.04 (0.73 to 1.49) | 0.81 | 0.97 (0.68 to 1.39) | 0.87 |
| Race: Hispanic | 1.3 (0.96 to 1.77) | 0.09 | 1.22 (0.89 to 1.66) | 0.22 |
| Race: White | 0.96 (0.71 to 1.29) | 0.78 | 0.95 (0.71 to 1.28) | 0.75 |
| CCI: 1-2 | 1.27 (1.05 to 1.53) | 0.01 | 0.95 (0.72 to 1.25) | 0.71 |
| CCI: 3-4 | 1.12 (0.83 to 1.51) | 0.47 | 0.85 (0.58 to 1.25) | 0.4 |
| CCI: ≥5 | 1.48 (1.08 to 2.03) | 0.02 | 1.12 (0.73 to 1.73) | 0.6 |
| Overweight | 1 (0.79 to 1.27) | 0.99 | 0.96 (0.75 to 1.23) | 0.74 |
| Obese | 1.22 (0.95 to 1.57) | 0.12 | 1.11 (0.86 to 1.44) | 0.42 |
| Severely Obese | 1.55 (1.2 to 2.01) | <0.01 | 1.32 (1 to 1.73) | 0.05 |
| mRNA vaccine receipt | 0.66 (0.54 to 0.81) | <0.01 | 0.72 (0.59 to 0.89) | <0.01 |
| Days post-symptom onset | 1 (0.99 to 1.02) | 0.77 | 0.99 (0.98 to 1.01) | 0.56 |
| AIC |  |  | 2042.50 |  |
| BIC |  |  | 2130.04 |  |
| Num. obs. | 1273 |  | 1273 |  |
| *^a^Adjusted for age group, sex, race, comorbidity, obesity, vaccine receipt, and days post symptom onset* | | | | |
| *^b^Ref: Nasal Cluster* |  |  |  |  |
| *CCI = Charlson Comorbidity Index* | |  |  |  |
| *mRNA vaccines = Pfizer/BioNTech-BNT162b2, Moderna mRNA‐1273* | | | | |

**S5 Table.** **Clinical and demographic characteristics of 529 military health system beneficiaries who filled out surveys at six-months post-symptom onset by identified clusters**

|  | **Nasal cluster (N=160)** | **Sensory cluster (N=200)** | **Respiratory/ Systemic cluster (N=169)** | **Total (N=529)** | ***P* value*^a^*** |
| --- | --- | --- | --- | --- | --- |
| **Moderate to severe symptoms at six-months** | | |  |  | 0.02 |
| No | 154 (96.2%) | 176 (88.0%) | 150 (88.8%) | 480 (90.7%) |  |
| Yes | 6 (3.8%) | 24 (12.0%) | 19 (11.2%) | 49 (9.3%) |  |
| **Age group** |  |  |  |  | < 0.01 |
| 18-44 | 85 (53.1%) | 131 (65.5%) | 77 (45.6%) | 293 (55.4%) |  |
| 45-64 | 45 (28.1%) | 60 (30.0%) | 70 (41.4%) | 175 (33.1%) |  |
| 65+ | 30 (18.8%) | 9 (4.5%) | 22 (13.0%) | 61 (11.5%) |  |
| **Gender** |  |  |  |  | < 0.01 |
| Female | 64 (40.0%) | 105 (52.5%) | 64 (37.9%) | 233 (44.0%) |  |
| Male | 96 (60.0%) | 95 (47.5%) | 105 (62.1%) | 296 (56.0%) |  |
| **Race/Ethnicity** |  |  |  |  | < 0.01 |
| Black | 22 (13.8%) | 23 (11.5%) | 21 (12.4%) | 66 (12.5%) |  |
| Hispanic or Latino | 23 (14.4%) | 51 (25.5%) | 55 (32.5%) | 129 (24.4%) |  |
| Other | 13 (8.1%) | 15 (7.5%) | 17 (10.1%) | 45 (8.5%) |  |
| White | 102 (63.8%) | 111 (55.5%) | 76 (45.0%) | 289 (54.6%) |  |
| **Charlson Comorbidity Index** | | |  |  | < 0.01 |
| 0 | 85 (53.1%) | 143 (71.5%) | 79 (46.7%) | 307 (58.0%) |  |
| 1-2 | 41 (25.6%) | 36 (18.0%) | 55 (32.5%) | 132 (25.0%) |  |
| 3-4 | 24 (15.0%) | 14 (7.0%) | 22 (13.0%) | 60 (11.3%) |  |
| >5 | 10 (6.2%) | 7 (3.5%) | 13 (7.7%) | 30 (5.7%) |  |
| **Severity** |  |  |  |  | < 0.01 |
| Hospitalized | 24 (15.0%) | 22 (11.0%) | 73 (43.2%) | 119 (22.5%) |  |
| Outpatient | 136 (85.0%) | 178 (89.0%) | 96 (56.8%) | 410 (77.5%) |  |
| **Variants** |  |  |  |  | 0.24 |
| Alpha | 8 (5.0%) | 6 (3.0%) | 13 (7.7%) | 27 (5.1%) |  |
| Delta | 29 (18.1%) | 39 (19.5%) | 29 (17.2%) | 97 (18.3%) |  |
| Others***^b^*** | 78 (48.8%) | 93 (46.5%) | 90 (53.3%) | 261 (49.3%) |  |
| Unknown | 45 (28.1%) | 62 (31.0%) | 37 (21.9%) | 144 (27.2%) |  |
| **BMI** |  |  |  |  | 0.01 |
| Normal | 35 (21.9%) | 48 (24.0%) | 21 (12.4%) | 104 (19.7%) |  |
| Overweight | 59 (36.9%) | 70 (35.0%) | 53 (31.4%) | 182 (34.4%) |  |
| Obese | 33 (20.6%) | 52 (26.0%) | 59 (34.9%) | 144 (27.2%) |  |
| Severely Obese | 33 (20.6%) | 30 (15.0%) | 36 (21.3%) | 99 (18.7%) |  |
| **Military Status** |  |  |  |  | < 0.01 |
| Active duty | 76 (47.5%) | 107 (53.5%) | 60 (35.5%) | 243 (45.9%) |  |
| Dependent | 41 (25.6%) | 65 (32.5%) | 41 (24.3%) | 147 (27.8%) |  |
| Retired military | 43 (26.9%) | 28 (14.0%) | 68 (40.2%) | 139 (26.3%) |  |
| **Department of Defense affiliation** | |  |  |  | < 0.01 |
| Air Force | 32 (20.0%) | 22 (11.0%) | 35 (20.7%) | 89 (16.8%) |  |
| Army | 75 (46.9%) | 94 (47.0%) | 76 (45.0%) | 245 (46.3%) |  |
| Coast Guard | 0 (0.0%) | 0 (0.0%) | 4 (2.4%) | 4 (0.8%) |  |
| Marines | 8 (5.0%) | 4 (2.0%) | 14 (8.3%) | 26 (4.9%) |  |
| Navy | 39 (24.4%) | 76 (38.0%) | 36 (21.3%) | 151 (28.5%) |  |
| Other*^d^* | 6 (3.8%) | 4 (2.0%) | 4 (2.4%) | 14 (2.6%) |  |
| **Vaccine breakthrough cases** | |  |  |  | 0.02 |
| No | 111 (69.4%) | 152 (76.0%) | 139 (82.2%) | 402 (76.0%) |  |
| Yes | 49 (30.6%) | 48 (24.0%) | 30 (17.8%) | 127 (24.0%) |  |
| **Vaccine** |  |  |  |  | < 0.01 |
| Pfizer | 41 (25.6%) | 53 (26.5%) | 33 (19.5%) | 127 (24.0%) |  |
| Moderna | 14 (8.8%) | 5 (2.5%) | 6 (3.6%) | 25 (4.7%) |  |
| Unvaccinated | 105 (65.6%) | 142 (71.0%) | 130 (76.9%) | 377 (71.3%) |  |
| *^a^n x k Fisher’s exact test* | |  |  |  |  |
| *^b^Other variants: Beta, Eta, Epsilon, Gamma, Iota, Theta* | | | | | |
| *^c^Sequence failed to be mapped to a lineage* | | | | | |
| *^d^Other DOD affiliation includes National Guard, National Oceanic and Atmospheric Administration, US Public Health Service, and missing affiliations.* | | | | | |

| **S6 Table. Prolonged COVID-19 symptoms of 529 Military Health System beneficiaries by identified clusters at six-months** | | | | |
| --- | --- | --- | --- | --- |
|  | **Nasal cluster (N=160)** | **Sensory cluster (N=200)** | **Respiratory/Systemic cluster (N=169)** | **Total (N=529)** |
| **Fatigue** |  |  |  |  |
| Yes | 1 (0.6%) | 11 (5.5%) | 10 (5.9%) | 22 (4.2%) |
| **Loss of sense of smell and/or taste** | | | |  |
| Yes | 3 (1.9%) | 14 (7.0%) | 4 (2.4%) | 21 (4.0%) |
| **Dyspnea (Difficulty breathing / shortness of breath)** | | | |  |
| Yes | 1 (0.6%) | 10 (5.0%) | 9 (5.3%) | 20 (3.8%) |
| **Exercise intolerance** | |  |  |  |
| Yes | 1 (0.6%) | 8 (4.0%) | 9 (5.3%) | 18 (3.4%) |
| **Headache** |  |  |  |  |
| Yes | 2 (1.2%) | 4 (2.0%) | 9 (5.3%) | 15 (2.8%) |
| **Joint pain** |  |  |  |  |
| Yes | 1 (0.6%) | 5 (2.5%) | 8 (4.7%) | 14 (2.6%) |
| **Changes in ability to think** | |  |  |  |
| Yes | 1 (0.6%) | 4 (2.0%) | 7 (4.1%) | 12 (2.3%) |
| **Change in ability to move** | |  |  |  |
| Yes | 1 (0.6%) | 1 (0.5%) | 3 (1.8%) | 5 (0.9%) |
| **Wheezing** |  |  |  |  |
| Yes | 0 (0.0%) | 3 (1.5%) | 2 (1.2%) | 5 (0.9%) |
| **Cough** |  |  |  |  |
| Yes | 1 (0.6%) | 1 (0.5%) | 2 (1.2%) | 4 (0.8%) |
| **Discoloration of fingers/toes** | |  |  |  |
| Yes | 0 (0.0%) | 1 (0.5%) | 1 (0.6%) | 2 (0.4%) |
| **Changes in level of consciousness (coma, lethargy)** | | | |  |
| Yes | 0 (0.0%) | 0 (0.0%) | 1 (0.6%) | 1 (0.2%) |
|  |  |  |  |  |

| **S7 Table: Univariable and multivariable models to identify correlates of long COVID among those reported persistent moderate to severe symptoms at six months** | | | | | |
| --- | --- | --- | --- | --- | --- |
| **Covariates** | **Unadjusted risk ratio (95% CI)** | | ***P* value** | **Adjusted risk ratio^a^ (95% CI)** | ***P* value** |
| Sensory Cluster^b^ | | 3.2 (1.31 to 7.83) | 0.01 | 2.86 (1.14 to 7.18) | 0.03 |
| Respiratory/Systemic Cluster^b^ | | 3 (1.2 to 7.51) | 0.02 | 2.89 (1.12 to 7.44) | 0.03 |
| Age group: 45-64 | 0.84 (0.45 to 1.56) | | 0.57 | 0.83 (0.34 to 2.02) | 0.68 |
| Age group: 65+ | 0.64 (0.23 to 1.82) | | 0.4 | 0.64 (0.15 to 2.75) | 0.54 |
| Sex: Male | 0.7 (0.4 to 1.22) | | 0.21 | 0.73 (0.41 to 1.29) | 0.28 |
| Race: Black | 0.68 (0.17 to 2.73) | | 0.59 | 0.7 (0.17 to 2.83) | 0.61 |
| Race: Hispanic | 1.13 (0.37 to 3.48) | | 0.83 | 1.03 (0.33 to 3.22) | 0.96 |
| Race: White | 1.09 (0.38 to 3.11) | | 0.87 | 1.29 (0.44 to 3.73) | 0.64 |
| CCI: 1-2 | 0.68 (0.32 to 1.42) | | 0.3 | 0.73 (0.27 to 1.96) | 0.53 |
| CCI: 3-4 | 0.83 (0.32 to 2.12) | | 0.69 | 1.06 (0.31 to 3.67) | 0.93 |
| CCI: ≥5 | 1.32 (0.47 to 3.74) | | 0.6 | 1.66 (0.43 to 6.35) | 0.46 |
| Overweight | 0.93 (0.38 to 2.24) | | 0.87 | 0.95 (0.39 to 2.31) | 0.9 |
| Obese | 1.53 (0.66 to 3.56) | | 0.32 | 1.58 (0.66 to 3.76) | 0.3 |
| Severely obese | 1.44 (0.58 to 3.59) | | 0.43 | 1.57 (0.6 to 4.06) | 0.36 |
| mRNA vaccine receipt | 0.41 (0.19 to 0.92) | | 0.03 | 0.43 (0.19 to 0.97) | 0.04 |
| Days post-symptom onset | 0.98 (0.93 to 1.04) | | 0.55 | 0.98 (0.92 to 1.04) | 0.45 |
| AIC |  | |  | 343.45 |  |
| BIC |  | |  | 416.14 |  |
| Num. obs. | 529 | |  | 529 |  |
| *^a^Adjusted for age group, sex, race, comorbidity, obesity, vaccine receipt, and days post symptom onset* | | | | | |
| *^b^Ref: Nasal Cluster* |  | |  |  |  |
| *CCI = Charlson Comorbidity Index* | | |  |  |  |
| *mRNA vaccines = Pfizer/BioNTech-BNT162b2, Moderna mRNA‐1273* | | | | |  |

| **S8 Table: Univariable and multivariable models to compare adjusted log10 based CRP levels in identified clusters** | | | | |
| --- | --- | --- | --- | --- |
| **Covariates** | **Coefficients**  **(95% CI)** | ***P* value** | **Adjusted Coefficients^a^**  **(95% CI)** | ***P* value** |
| Sensory Cluster^b^ | 0.09 (-0.09 to 0.28) | 0.32 | 0.1 (-0.07 to 0.27) | 0.26 |
| Respiratory/Systemic Cluster^b^ | 0.4 (0.22 to 0.58) | <0.01 | 0.25 (0.09 to 0.42) | <0.01 |
| Age group: 45-64 | 0.56 (0.41 to 0.72) | <0.01 | 0.3 (0.09 to 0.51) | 0.01 |
| Age group: 65+ | 0.54 (0.29 to 0.78) | <0.01 | 0.38 (0.04 to 0.71) | 0.03 |
| Sex: Male | -0.02 (-0.17 to 0.13) | 0.77 | -0.06 (-0.2 to 0.07) | 0.35 |
| Race: Black | 0.02 (-0.28 to 0.33) | 0.88 | -0.11 (-0.38 to 0.17) | 0.45 |
| Race: Hispanic | 0.22 (-0.05 to 0.5) | 0.11 | 0.14 (-0.11 to 0.38) | 0.27 |
| Race: White | -0.17 (-0.43 to 0.09) | 0.2 | -0.11 (-0.35 to 0.13) | 0.37 |
| CCI: 1-2 | 0.57 (0.41 to 0.74) | <0.01 | 0.19 (-0.03 to 0.41) | 0.09 |
| CCI: 3-4 | 0.42 (0.17 to 0.68) | <0.01 | 0.08 (-0.23 to 0.39) | 0.62 |
| CCI: ≥5 | 0.61 (0.31 to 0.92) | <0.01 | 0.2 (-0.18 to 0.57) | 0.3 |
| Overweight | 0.46 (0.27 to 0.66) | <0.01 | 0.35 (0.16 to 0.54) | <0.01 |
| Obese | 0.7 (0.49 to 0.9) | <0.01 | 0.52 (0.32 to 0.72) | <0.01 |
| Severely obese | 1.08 (0.85 to 1.3) | <0.01 | 0.81 (0.58 to 1.03) | <0.01 |
| mRNA vaccine receipt | -0.54 (-0.73 to -0.34) | <0.01 | -0.32 (-0.51 to -0.14) | <0.01 |
| Sampling time | -<0.01 (-0.02 to 0.01) | 0.89 | -0.01 (-0.02 to <0.01) | 0.09 |
| *Number of observations: 607* | |  |  |  |
| *^a^Adjusted for age group, sex, race, comorbidity, obesity, vaccine receipt, and days post symptom onset* | | | | |
| *^b^Ref: Nasal Cluster* | |  |  |  |
| *CCI = Charlson Comorbidity Index* | |  |  |  |
| *mRNA vaccines = Pfizer/BioNTech-BNT162b2, Moderna mRNA‐1273* | | | |  |

| **S9 Table: Univariable and multivariable models to compare adjusted log10 based IL-6 levels in identified clusters** | | | | |
| --- | --- | --- | --- | --- |
| **Covariates** | **Coefficients (95% CI)** | ***P* value** | **Adjusted Coefficients^a^**  **(95% CI)** | ***P* value** |
| Sensory Cluster^b^ | -0.01 (-0.12 to 0.1) | 0.87 | 0.06 (-0.04 to 0.16) | 0.27 |
| Respiratory/Systemic Cluster^b^ | 0.23 (0.12 to 0.34) | <0.01 | 0.16 (0.06 to 0.26) | <0.01 |
| Age group: 45-64 | 0.42 (0.33 to 0.51) | <0.01 | 0.21 (0.08 to 0.33) | <0.01 |
| Age group: 65+ | 0.53 (0.39 to 0.67) | <0.01 | 0.25 (0.05 to 0.45) | 0.01 |
| Sex: Male | 0.06 (-0.03 to 0.15) | 0.19 | 0.03 (-0.05 to 0.11) | 0.5 |
| Race: Black | 0.01 (-0.17 to 0.2) | 0.9 | -0.1 (-0.26 to 0.06) | 0.22 |
| Race: Hispanic | -0.04 (-0.21 to 0.12) | 0.61 | -0.1 (-0.25 to 0.04) | 0.16 |
| Race: White | -0.16 (-0.31 to 0) | 0.05 | -0.18 (-0.31 to -0.04) | 0.01 |
| CCI: 1-2 | 0.38 (0.28 to 0.48) | <0.01 | 0.14 (0.01 to 0.27) | 0.03 |
| CCI: 3-4 | 0.47 (0.32 to 0.62) | <0.01 | 0.25 (0.07 to 0.43) | 0.01 |
| CCI: ≥5 | 0.61 (0.44 to 0.78) | <0.01 | 0.36 (0.14 to 0.58) | <0.01 |
| Overweight | 0.2 (0.08 to 0.32) | <0.01 | 0.14 (0.03 to 0.25) | 0.01 |
| Obese | 0.38 (0.25 to 0.5) | <0.01 | 0.26 (0.14 to 0.38) | <0.01 |
| Severely obese | 0.59 (0.45 to 0.73) | <0.01 | 0.44 (0.31 to 0.57) | <0.01 |
| mRNA vaccine receipt | -0.13 (-0.25 to -0.01) | 0.04 | 0.03 (-0.08 to 0.14) | 0.58 |
| Sampling time | 0.01 (0 to 0.02) | 0.05 | <0.01 (<0.01 to 0.01) | 0.31 |
| *Number of observations: 607* | |  |  |  |
| *^a^Adjusted for age group, sex, race, comorbidity, obesity, vaccine receipt, and days post symptom onset* | | | | |
| *^b^Ref: Nasal Cluster* | |  |  |  |
| *CCI = Charlson Comorbidity Index* | |  |  |  |
| *mRNA vaccines = Pfizer/BioNTech-BNT162b2, Moderna mRNA‐1273* | | | | |
