# Supplementary material for: A machine learning approach identifies distinct early-symptom cluster phenotypes which correlate with hospitalization, failure to return to activities, and prolonged COVID-19 symptoms
Source: PLoS One. 2023 Feb 9;18(2):e0281272. doi: 10.1371/journal.pone.0281272 (PMC9910657; doi:10.1371/journal.pone.0281272)
Supplement: Attachment — Submitted filename: Submitted filename: Response to Reviewer PONE-D-22-31484 010523.docx [file pone.0281272.s002.docx]

January 5, 2023

Gayathri Delanerolle, MBBS, BSc, MSc

Academic Editor, *PLOS ONE*

Dear Dr. Delanerolle,

Thank you for the ongoing consideration of this manuscript. We are re-submitting our revised manuscript PONE-D-22-31484 by Epsi et al. entitled “*A machine learning approach identifies distinct early-symptom cluster phenotypes which correlate with hospitalization, failure to return to activities, and prolonged COVID-19 symptoms*”.

We sincerely appreciate the academic editor and Reviewer 1’s valuable comments and suggestions. We agree these improve the quality of the manuscript and have revised the manuscript accordingly. Our revisions also comply with the format requirements of *PLOS ONE*. Minor grammar changes have been made elsewhere to accommodate important requested additional discussion within the context of the word limits.

Specific responses to the academic editor comments are below:

**Comment:** **Please ensure that your manuscript meets PLOS ONE's style requirements, including those for file naming. The PLOS ONE style templates can be found at https://journals.plos.org/plosone/s/file?id=wjVg/PLOSOne_formatting_sample_main_body.pdf and https://journals.plos.org/plosone/s/file?id=ba62/PLOSOne_formatting_sample_title_authors_affiliations.pdf**

**Response:** We appreciate the academic editor’s careful evaluation of our study. We have revised the manuscript and complied using PLOS ONE’s style requirement.

**Comment:** **Please amend your current ethics statement to address the following concerns:**

**a) Did participants provide their written or verbal informed consent to participate in this study?**

**b) If consent was verbal, please explain**

**i) why written consent was not obtained,**

**ii) how you documented participant consent, and**

**iii) whether the ethics committees/IRB approved this consent procedure.**

**Response:** We added a statement in the method section:

*This study was approved by the Uniformed Services University of the Health Sciences (USUHS) Institutional Review Board (IRB) under protocol IDCRP-085 [22]; all participants or their legally authorized representative provided informed consent to participate.*

**Comment: Thank you for stating the following in the Competing Interests section:**

**"*****Potential conflicts of interest. S. D. P., T. H. B, and M.P.S. report that the Uniformed Services University (USU) Infectious Diseases Clinical Research Program (IDCRP), a US Department of Defense institution, and the Henry M. Jackson Foundation (HJF) were funded under a Cooperative Research and Development Agreement to conduct an unrelated phase III COVID-19 monoclonal antibody immunoprophylaxis trial sponsored by AstraZeneca. The HJF, in support of the USU IDCRP, was funded by the Department of Defense Joint Program Executive Office for Chemical, Biological, Radiological, and Nuclear Defense to augment the conduct of an unrelated phase III vaccine trial sponsored by AstraZeneca. Both of these trials were part of the US Government COVID-19 response. Neither is related to the work presented here*"**

**Please confirm that this does not alter your adherence to all PLOS ONE policies on sharing data and materials, by including the following statement: "This does not alter our adherence to PLOS ONE policies on sharing data and materials.” (as detailed online in our guide for authors** [**http://journals.plos.org/plosone/s/competing-interests**](http://journals.plos.org/plosone/s/competing-interests)**). If there are restrictions on sharing of data and/or materials, please state these. Please note that we cannot proceed with consideration of your article until this information has been declared.**

**Please include your updated Competing Interests statement in your cover letter; we will change the online submission form on your behalf.**

**Response:** We thank the academic editor; we updated our Competing Interests statement as follows:

**“***Potential conflicts of interest. S. D. P., T. H. B, D.R.T, and M.P.S. report that the Uniformed Services University (USU) Infectious Diseases Clinical Research Program (IDCRP), a US Department of Defense institution, and the Henry M. Jackson Foundation (HJF) were funded under a Cooperative Research and Development Agreement to conduct an unrelated phase III COVID-19 monoclonal antibody immunoprophylaxis trial sponsored by AstraZeneca. The HJF, in support of the USU IDCRP, was funded by the Department of Defense Joint Program Executive Office for Chemical, Biological, Radiological, and Nuclear Defense to augment the conduct of an unrelated phase III vaccine trial sponsored by AstraZeneca. Both of these trials were part of the US Government COVID-19 response. Neither is related to the work presented here. This does not alter our adherence to PLOS ONE policies on sharing data and materials.”*

**Comment: In your Data Availability statement, you have not specified where the minimal data set underlying the results described in your manuscript can be found. PLOS defines a study's minimal data set as the underlying data used to reach the conclusions drawn in the manuscript and any additional data required to replicate the reported study findings in their entirety. All PLOS journals require that the minimal data set be made fully available. For more information about our data policy, please see http://journals.plos.org/plosone/s/data-availability.**

**""Upon re-submitting your revised manuscript, please upload your study’s minimal underlying data set as either Supporting Information files or to a stable, public repository and include the relevant URLs, DOIs, or accession numbers within your revised cover letter. For a list of acceptable repositories, please see http://journals.plos.org/plosone/s/data-availability#loc-recommended-repositories. Any potentially identifying patient information must be fully anonymized.**

**Important: If there are ethical or legal restrictions to sharing your data publicly, please explain these restrictions in detail. Please see our guidelines for more information on what we consider unacceptable restrictions to publicly sharing data: http://journals.plos.org/plosone/s/data-availability#loc-unacceptable-data-access-restrictions. Note that it is not acceptable for the authors to be the sole named individuals responsible for ensuring data access.**

**We will update your Data Availability statement to reflect the information you provide in your cover letter.**

**Response:** We thank the academic editor, we have updated our Data Availability statement as follows,

“*Data for this study are available from the Infectious Disease Clinical Research Program (IDCRP), headquartered at the Uniformed Services University of the Health Sciences (USU), Department of Preventive Medicine and Biostatistics. Review by the USU Institutional Review Board is required for use of the data collected under this protocol. Furthermore, the data set includes Military Health System data collected under a Data Use Agreement that requires accounting for uses of the data. Data requests may be sent to: Address: 6270A Rockledge Drive, Suite 250, Bethesda, MD 20817. Email: contactus@idcrp.org.”*

**Comment:** **One of the noted authors is a group or consortium "EPICC COVID-19 Cohort Study Group". In addition to naming the author group, please list the individual authors and affiliations within this group in the acknowledgments section of your manuscript. Please also indicate clearly a lead author for this group along with a contact email address.**

**Response:** We have revised the group author list and updated in the Acknowledgement section:

“*We sincerely thank the members of the EPICC COVID-19 Cohort Study Group for their many contributions in conducting the study and ensuring effective protocol operations. The following members were all closely involved with the design, implementation, and oversight of the study and have met group authorship criteria for this manuscript:*

*Brooke Army Medical Center, Fort Sam Houston, TX: Jessica J. Cowden; Teresa M. Merritt*

*Fort Belvoir Community Hospital, Fort Belvoir, VA: Nora Elnahas; Christa Glinn; Donna Jennings; Chiquita West*

*ACESO, Henry M. Jackson Foundation, Inc., Bethesda, MD: Danielle Clark*

*Madigan Army Medical Center, Joint Base Lewis McChord, WA: Susan Chambers; Cristin A. Mount*

*Naval Medical Center San Diego, San Diego, CA: Nichol M. Kirkland*

*Tripler Army Medical Center, Honolulu, HI: Catherine Uyehara*

*Uniformed Services University of the Health Sciences, Bethesda, MD: Heidi Adams; Celia Byrne; Mark Fritschlanski; Edward Parmelee; Jennifer Rusiecki; Emily Samuels; Ann Scher; Melissa Wayman*

*United States Air Force School of Aerospace Medicine, Dayton, OH: Richard Chapleau; Monica Christian; Kelsey Lanter; Elizabeth Macias*

*United States Coast Guard, Washington, DC: John K. Iskander*

*Womack Army Medical Center, Fort Bragg, NC: Kathryn J. Lago*”

**Comment:** **Please include captions for your Supporting Information files at the end of your manuscript, and update any in-text citations to match accordingly. Please see our Supporting Information guidelines for more information: http://journals.plos.org/plosone/s/supporting-information.**

**Response:** We have added the supporting information files details at the end of the manuscript.

**Comment:** **Please review your reference list to ensure that it is complete and correct. If you have cited papers that have been retracted, please include the rationale for doing so in the manuscript text, or remove these references and replace them with relevant current references. Any changes to the reference list should be mentioned in the rebuttal letter that accompanies your revised manuscript. If you need to cite a retracted article, indicate the article’s retracted status in the References list and also include a citation and full reference for the retraction notice.**

**Response:** We have carefully reviewed the reference list and ensure that it is complete and correct.

Specific responses to the reviewer comments are below:

**Reviewer #1:**

**Comment:** **Authors have presented an important piece of information in understanding symptom patterns of the Covid-19 and likely clinical course. I have few suggestions for consideration of the authors**

**1. It will be helpful for international readers if it is clarified that the beneficiaries included/not included the dependent family members of the military. Also, in the table 1, the occupation might be helpful, as a predominant in-service participants might have a different level of pre-illness physical fitness**

**Response:** We appreciate the reviewer’s evaluation of our manuscript and thank the reviewer for this important point. We have updated and added rows to Table 1 indicating the military status and affiliation of the study participants.

**2. It is also noted that only half of the enrolled participants completed FLU-PRO Plus survey, and further around half of these participants responded to the survey at six month. Therefore, authors may present some demographic of all enrolled participants and clinical characteristics of participants who completed the first survey, for benefit of readers to be able to compare the participating and non-participating enrollees. This may be added as supplement.**

**Response:** We apologies for any confusion this may have caused. Upon rereading our manuscript, we realize we did not explain the selection process clearly enough. We have revised the relevant parts of the manuscript to clarify this point in the method section:

*“Along with FLU-PRO Plus survey, EPICC participants were also requested to complete online follow-up* surveys that included questions about presence and severity of ongoing symptoms *at 1, 3, 6, 9, and 12 months. Participants who responded at approximately six months post-symptom onset (135-224 days) were included in the prolonged COVID-19 symptoms analysis (Supplementary Figure 1).”*

Further, we added a table with clinical and demographic characteristics of subset participants who filled out follow-up surveys at six months post-symptom onset (S5 Table).

We have compared the responder and non-responder groups in the analysis. In the Result section, we have included a sentence discussing this comparison.

*“In the non-responder group (those who did not filled out FLU-PRO Plus survey, N = 433), which was excluded from the analysis, consisted of approximately 20% children, 29.3% hospitalized individuals, and 13.7% tested negative for SARS-CoV-2. Only 2 participants reported moderate to severe symptoms at 6 months, and overall, the group dominantly exhibits dependent (42.0%) than the responder group (S1 Table).”*

We have also added a supplementary table (S1 Table) to provide further details on this responder and non-responder comparison.

We hope that these changes will make the information clearer and easier to understand. We believe that our comments address the suggestions and concerns raised by the Reviewer, and hope that this revised manuscript will be suitable for publication in *PLOS ONE*.

Thank you for your careful consideration.

Respectfully,

Stephanie A. Richard, PhD

Epidemiologist / Biostatistician

HJF for the Infectious Disease Clinical Research Program

Department of Preventive Medicine and Biostatistics

Uniformed Services University of the Health Sciences

6720A Rockledge Drive, Suite 250

Rockville, MD 20817

Phone: 202-674-5532

Email: srichard@idcrp.org
